# *Melissa officinalis* Acidic Fraction Protects Cultured Cerebellar Granule Neurons Against Beta Amyloid-Induced Apoptosis and Oxidative Stress

**DOI:** 10.22074/cellj.2016.4722

**Published:** 2016-09-26

**Authors:** Maliheh Soodi, Abolfazl Dashti, Homa Hajimehdipoor, Shole Akbari, Nasim Ataei

**Affiliations:** 1 Department of Toxicology, Faculty of Medical Sciences, Tarbiat Modares University, Tehran, Iran; 2 Department of Traditional Pharmacy, School of Traditional Medicine, Shahid Beheshti University of Medical Sciences, Tehran, Iran

**Keywords:** *Melissa officinalis*, Nicotinic Receptor, Apoptosis, Alzheimer’s Disease

## Abstract

**Objective:**

Extracellular deposition of the beta-amyloid (Aβ) peptide, which is the main finding in the pathophysiology of Alzheimer’s disease (AD), leads to oxidative damage and apoptosis in neurons. *Melissa officinalis (M. officinalis)* is a medicinal plant from the Lamiaceae family that has neuroprotective activity. In the present study we have investigated the protective effect of the acidic fraction of *M. officinalis* on Aβ-induced oxidative stress and apoptosis in cultured cerebellar granule neurons (CGN). Additionally, we investigated a possible role of the nicotinic receptor.

**Materials and Methods:**

This study was an *in vitro* experimental study performed on mice cultured CGNs. CGNs were pre-incubated with different concentrations of the acidic fraction of *M. officinalis* for 24 hours, followed by incubation with Aβ for an additional 48 hours. CGNs were also pre-incubated with the acidic fraction of *M. officinalis* and mecamylamin, followed by incubation with Aβ. We used the 3-(4,5-dimethylthiazol-2-yl)-2,5- diphenyltetrazolium bromide (MTT) assay to measure cell viability. Acetylcholinesterase (AChE) activity, reactive oxygen species (ROS) production, lipidperoxidation, and caspase-3 activity were measured after incubation. Hochst/annexin Vfluorescein isothiocyanate (FITC)/propidium iodide (PI) staining was performed to detect apoptotic cells.

**Results:**

The acidic fraction could protect CGNs from Aβ-induced cytotoxicity. Mecamylamine did not abolish the protective effect of the acidic fraction. AChE activity, ROS production, lipid peroxidation, and caspase-3 activity increased after Aβ incubation. Preincubation with the acidic fraction of *M. officinalis* ameliorated these factors and decreased the number of apoptotic cells.

**Conclusion:**

Our results indicated that the protective effect of the acidic fraction of M. officinalis was not mediated through nicotinic receptors. This fraction could protect CGNs through antioxidant and anti-apoptotic activities.

## Introduction

Alzheimer’s disease (AD) is a progressive neurodegenerative disorder that causes memory deficits and can be fatal (1). Two hallmarks in the pathogenesis of AD are neurofibrillary tangles (NFT) and neuritic plaques. The aggregated fibrillar beta-amyloid (Aβ) peptide is the main component of extracellular plaques (2). Several proposed mechanisms describe the neurotoxicity of Aβ: production of reactive oxygen species (ROS) and oxidative stress; mitochondrial dysfunction and depletion of cellular ATP; increased intracellular calcium and excitotoxicity; and induction of inflammatory responses. These mechanisms result in synaptic dysfunction with neuronal loss through activation of apoptotic and necrotic cell death pathways (2). Several studies have reported that neuronal loss in AD occurs via a programmed cell death pathway (3,4). Evidence of apoptosis has been found in postmortem tissue from AD patients (5). It is postulated that inhibition of apoptotic processes can be an effective strategy in the prevention of AD (6). Apoptosis can be induced by ROS and oxidative stress produced by Aβ. Therefore, therapeutics that reduce free radicals or prevent their production can be beneficial for AD treatment. Hence, antioxidants are considered as one of therapeutic strategies to attenuate Aβ-induced apoptosis and ameliorate neurological outcome in AD (4). 

Because of the complex pathology of AD, new attempts intend to produce therapeutic agents that can stop disease progression through different pathways. Herbal medicines contain a number of components with different pharmacological effects (pro-cholinergic, antioxidant, anti-amyloid, and anti-inflammatory) and may be more effective in treatment of complex diseases (7). Recently, herbal treatments have been examined in animal and cellular models of AD. Clinical trials with AD subjects show beneficial effects of herbal medicine on cognitive functions and disease progression. 

*Melissa officinalis (M. officinalis, lemon balm)* is a medicinal plant from the Lamiaceae family. In Iran, this plant has been used as a folk medicine for numerous years (8). Medicinal preparations of this herb were used for treatment of indigestion, anemia, palpitations and mood disorders (9,10). *M. officinalis* impacts nervous disorders including reductions in excitability, anxiety and stress, and sleep disturbances (11). The total extract of *M. officinalis* and its different fractions have anticholinesterase activity (12). *M. officinalis* extract displays potent antioxidant activity and plant extracts can protect cells against oxidative damage induced by different pro-oxidant agents which eventually leads to lipid peroxidation (13). Administration of *M. officinalis* extract in AD patients can improve disease symptoms (14). The extract has been shown to alleviate scopolamine-induced amnesia in rats as an animal model of AD (15). However, the mechanism and constituents involved in these neuroprotective properties are not well known. In our previous study, we have reported that the acidic fraction of *M. officinalis* extract was more protective of PC12 cells against Aβ-induced toxicity compared to the total extract (16). It has been postulated that *M. officinalis* extract contains compounds with affinity for the nicotinic receptor (17). Several studies reported the role of the nicotinic receptor in Aβ-induced toxicity and nicotine attenuated Aβ-induced toxicity in cultured neuron (18). In the current study we intended to investigate the neuroprotective effect of the acidic fraction of *M. officinalis* extract against Aβ-induced oxidative stress and apoptosis in cultured cerebellar granule neurons (CGN). In addition, the role of the nicotinic receptor was investigated. 

## Materials and Methods

This study was an *in vitro* experimental study performed on cultured CGN obtained from mice. 

### Materials

Dulbecco’s modified Eagle’s Medium (DMEM), fetal bovine serum (FBS), penicillin-streptomycin (10000 U/mL), and trypsin (0.25%) were purchased from Gibco (USA). Poly-D-lysine (PDL) 

was purchased from Santa Cruz Biotechnology (USA). All other materials were purchased from Sigma (USA). 

### Primary culture of cerebellar granule neurons

Cerebellar granule cells (CGC) were obtained from the brains of 6-7 day-old BALB/c mice and cultured as described previously (19). The cerebella was dissected from early postnatal mice, digested with trypsin, and triturated to obtain a single cell suspension. Then, the cells were cultured on PDL-coated cell culture plates in DMEM that consisted of 10% FBS, 4.5 g/L glucose, 25 mM KCl, 100 mU/L, insulin and 1% v/v penicillin and streptomycin. Cells were maintained at 37˚C in a humidified atmosphere with 5% CO_2_ . Nonneuronal cell growth was inhibited by the addition of cytosine β-D-arabinofuranoside (Ara-C) to the cell culture medium at a final concentration of 20 µM, 48 hours after cell plating. We did not change the medium during the culture period. 7 days after plating (DIV 7), we evaluated the culture for neuronal growth *in vitro*. MAP2 protein immunostaining indicated that more than 95% of the cultured cells were neurons. Therefore, we used the DIV7 cells for the experiments. The Medical Ethics Committee of Tarbiat Modares University approved the procedures. 

### Plant material

The leaves of *M. officinalis* were collected from Gorgan (Golestan Province, Iran) in June 2012 and identified by M. Kamalinejad, a botanist from the Faculty of Pharmacy, Shahid Beheshti University of Medical Sciences (SBMU). A voucher specimen (No. 545) was kept in the Herbarium of the Faculty of Pharmacy, SBMU, Tehran, Iran. 

### Plant extraction

The total plant extract was obtained by the extraction of dried and milled plant leaves with 80% ethanol (1:10) according to the maceration method for 4 days. After each 24 hour period, the mixture was filtered and new solvent was added to the plant powder. The combined extracts were concentrated until dry. We prepared the acidic fraction according to a previously described method (16,20). 

### Treatment

CGNs were plated onto PDL-coated 96-well plates at 1×10^5^ cells/well. On DIV7, cells were pre-incubated with different concentrations (1-30 µg/ml) of the acidic fraction of *M. officinalis* for 24 hours then incubated with 20 μM of Aβ peptide for an additional 48 hours. Also cells were pre-incubated with 30 µg/ml of the acidic fraction of the extract plus mecamylamine as a nicotinic receptor antagonist. After 24 hours, we added Aβ (the final concentration was 20 μM) and the cells were incubated for an additional 48 hours. The stock solution of Aβ peptide (1 mM) was prepared by dissolving 1 mg Aβ peptide in 1 ml sterile deionized water. The solution was stored at -80˚C until use. Prior to use the Aβ peptide was aggregated by incubation at 37˚C for 3 days. Cell viability was measured by the 3-(4,5-dimethylthiazol-2-yl)-2,5diphenyltetrazolium bromide (MTT) assay. 

### 3-(4,5-dimethylthiazol-2-yl)-2,5-diphenyltetrazolium bromide (MTT) assay

The percent of surviving neurons was estimated by the MTT reduction assay. The culture medium was removed and replaced with medium that contained 0.5 mg/mL MTT reagent. After a 4-hour incubation period, the medium was replaced by 100 mL DMSO to dissolve the formazan crystals. Absorbance was measured at a wave length of 570 against 630 nm as the reference wavelength. Results are expressed as percentage of the control (21). 

### Lipid peroxidation measurement

Malondialdehyde (MDA), an indicator of lipid peroxidation, was measured by the thiobarbituric acid (TBA) assay. Cells were washed with PBS and subsequently harvested by trypsinization followed by sonication for 20 seconds. >One volume of cell lysate was mixed with two volumes of TBA reagent that contained 3.75% TCA and 0.0925% TBA. The mixture was incubated at 90˚C for 60 minutes. The cooled mixture was centrifuged at 1000 g for 10 minutes after which 200 µL from each standard or samples were transferred to a 96-well plate for fluorometric microplate reader analysis (22). The MDA standard curve was established using the stable MDA precursor, MDA-bis(dimethyl acetal). The amount of protein was measured by the Bradford method (23). 

### Acetylcholinesterase activity assay

Acetylcholinesterase (AChE) activity assays were carried out using an acetylthiocholine iodide (ATCh) substrate-based colorimetric method as described by Ellman et al. (24). The Ellman reagent (100 µL) that contained 100 mM NaHPO^4^ (pH=8.0), ATCh (75 mM as the substrate), and 10 mM 5,5´-Dithio-bis-(2nitrobenzoic acid) (DTNB) at a ratio of 100:2:5 was transferred to the 96-well microplate. Next, we added 50 µL cell lysate to the reaction mixture as an enzyme source. Absorbance was monitored at 412 nm during a 15 minute period. A blank that contained all components except for the cell lysate was run in parallel with the samples to omit spontaneous and non-enzymatic breakdown of ATCh. The reactions rate was subsequently calculated. The amount of protein was measured by the Bradford method. 

### Measurement of reactive oxygen species 

We evaluated ROS generation in the cells with a 2,7-dichlorodihydrofluorescein diacetate (DCFHDA) probe (25). After the incubation time, the medium was replaced by 10 µmol DCFH-DA that contained medium. After a 15 minute incubation period, the medium was removed. The cells were rinsed twice with Ca^2+^ -free PBS. The fluorescence intensity was measured by a fluorescence microplate reader (Biotek, USA) at an excitation wavelength of 488 nm and emission wavelength of 525 nm. 

### Caspase-3 activity assay

We have used the colorimetric assay to detect caspase-3 activity. In this assay, caspase-3 enzyme releases p-nitroaniline (pNA) from the ACDEVD-pNA substrate. The pNA absorbance was monitored at 405 nm. The cells were harvested by trypsinization, resuspended in 1 mL of cell lysis buffer (50 mM tris-HCl, pH=7.5, 1.0 mM DTT), sonicated for 20 seconds, and centrifuged at 1000 g for 10 minutes. The supernatant was collected for the caspase-3 assay. The AC-DEVD-pNA substrate (100 mM) stock solution was prepared in DMSO and further diluted by cell lysis buffer to make a 0.2 mM assay solution. We added 10 µl supernatant to the 90 µl assay solution and incubated the mixture at 37˚C for at least one hour. The absorbance was measured by a microplate reader at 405 nm (Biotek, USA) (26). 

### Hoechst/annexin V-fluorescein isothiocyanate/ propidium iodide staining

We used the Hoechst/annexin V-fluorescein isothiocyanate (FITC)/ propidium iodide (PI) triple staining detection kit to assess cell apoptosis. Annexin V-FITC, Hoechst 33258, and PI solutions were added to stain cells in the dark at room temperature for 15 minutes. Cells were subsequently washed with PBS (pH=7.2), fixed with 2 mL of 4% paraformaldehyde, and washed three times with PBS (pH=7.2), after which the wells were observed under a fluorescence microscope. 

### Statistical analysis

Data are presented as mean ± SEM of three separate experiments. Statistical analysis was performed by GraphPad Prism 5 software. Statistical differences were estimated by one-way ANOVA followed by the Newman-Keuls Multiple Comparison Test. P values of less than 0.05 were considered statistically significant. 

## Results

### Cell viability

**Fig.1:**
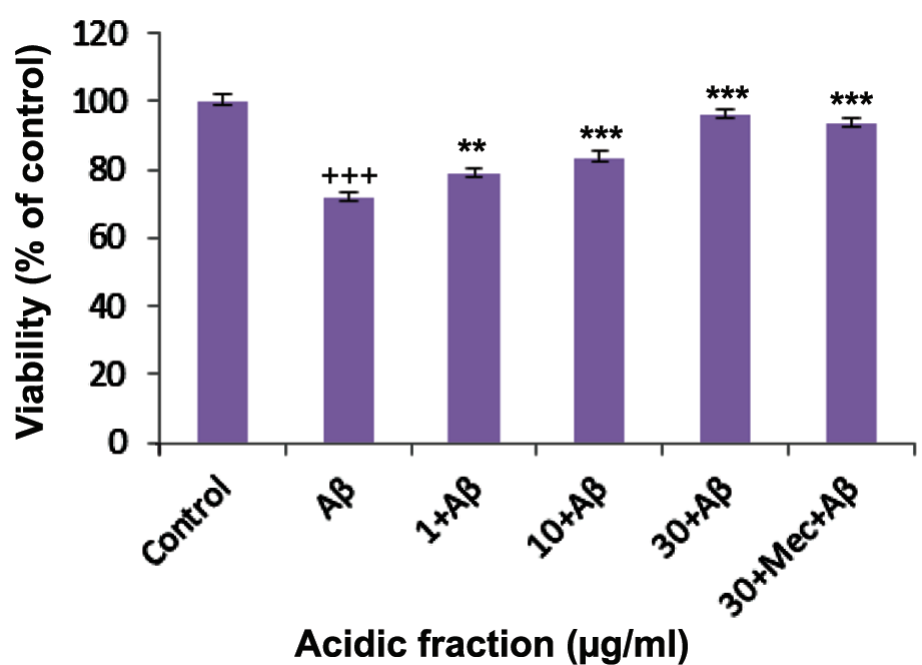
Effects of the *Melissa officinalis (M. officinalis)* acidic fraction on beta-amyloid (Aβ)-induced toxicity in cerebellar granule neurons (CGNs). Mec; Mecamylamine, +++; P<0.001 vs. control, **; P<0.01, and***; P<0.001 vs. Aβ-treated.

We used the MTT assay to evaluate the protective effect of the *M. officinalis* acidic fractions. CGNs incubated for 48 hours with Aβ had significantly reduced cell viability. Pretreatment of cells for 24 hours with different concentrations of the acidic fraction followed by addition of the Aβ peptide protected the CGNs from Aβ-induced toxicity (Fig.1). This effect was dose-dependent. We observed that 30 µg/ml of the acidic fraction completely reversed Aβ-induced toxicity and increased cell viability up to the control level. Mecamylamine could not abolish the protective effect of the extract. 

### Lipid peroxidation

**Fig.2:**
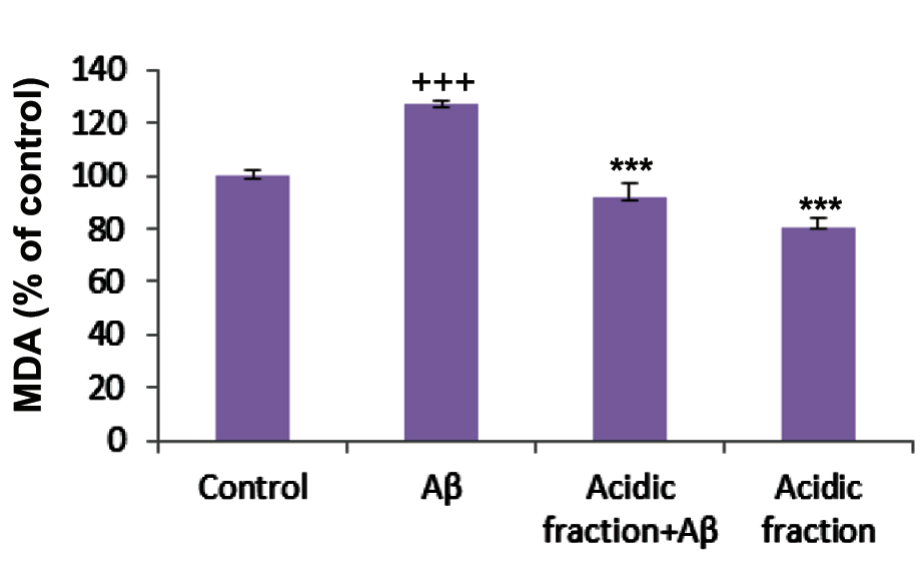
Protective effects of the *Melissa officinalis (M. officinalis)* acidic fraction on beta-amyloid (Aβ)-induced lipid peroxidation in cerebellar granule neurons (CGNs). MDA; Malondialdehyde, +++; P<0.001 vs. control, and ***; P<0.001 vs. Aβ-treated.

The amount of intracellular MDA, as a product of lipid peroxidation, significantly increased when cells were incubated for 48 hours with Aβ. Pretreatment of cells with 30 µg/ml of the acidic fraction for 24 hours followed by co-treatment with Aβ for 48 hours significantly attenuated Aβinduced MDA production (Fig.2). 

### Acetylcholinesterase activity

**Fig.3:**
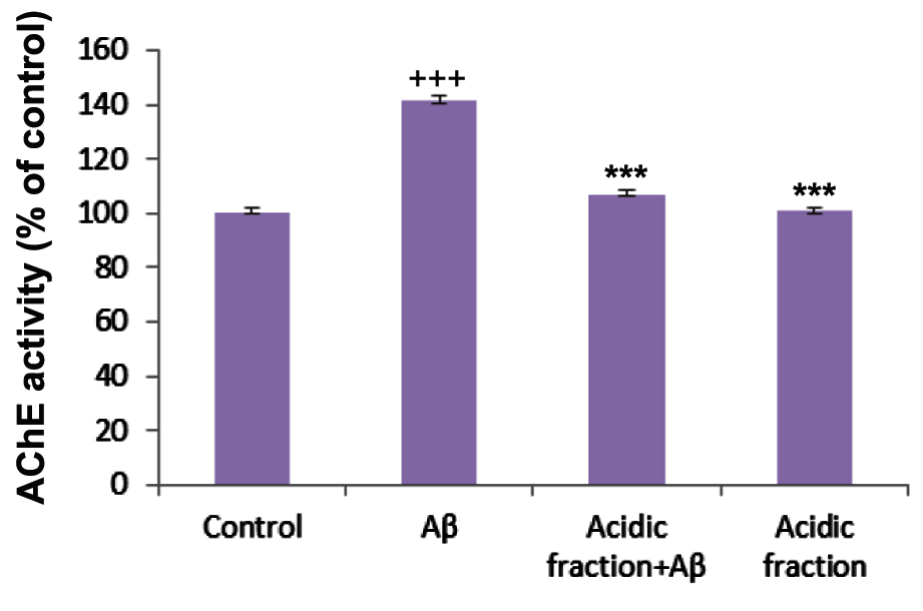
Acetylcholinesterase (AChE) enzyme activity in the studied groups. +++; P<0.001 vs. control and ***; P<0.001 vs. betaamyloid (Aβ)-treated.

After incubation of mature neurons for 48 hours with Aβ (20 µM), AChE activity significantly increased compared with the control group. Pretreatment followed by co-treatment with the acidic fraction attenuated the increase in Aβ-induced AChE activity (Fig.3). 

### Reactive oxygen species production

**Fig.4:**
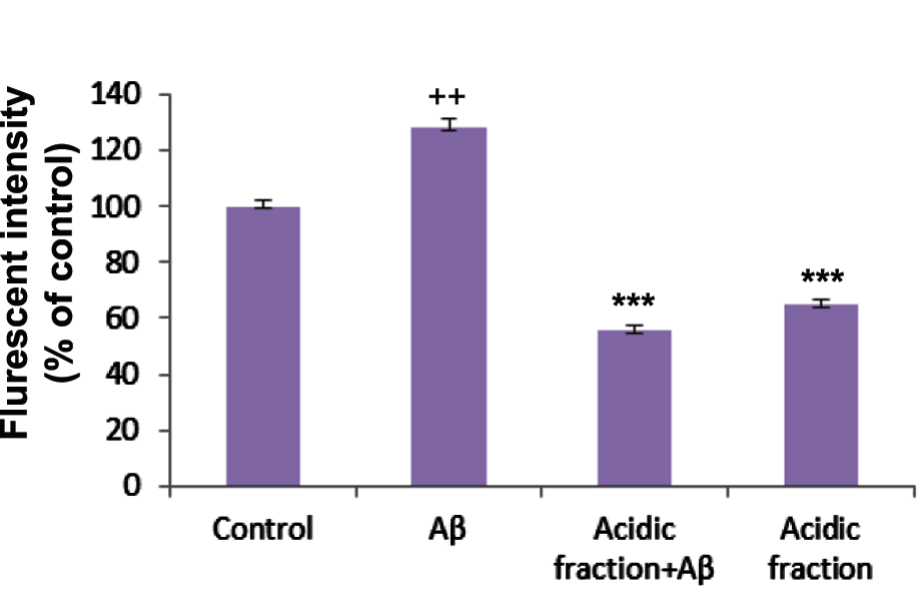
Protective effects of the *Melissa officinalis (M. officinalis)* acidic fraction on beta-amyloid (Aβ)-induced reactive oxygen species (ROS) production in cerebellar granule neurons (CGNs). ++; P<0.01 vs. control and ***; P<0.001 vs. Aβ-treated.

The amount of intracellular ROS significantly increased in cells incubated for 48 hours with Aβ. Cells pretreated with 30 μg/mL of the acidic fraction before the addition of Aβ showed lower mean fluorescence intensities compared to the Aβ treatment group (Fig.4). 

### Caspase-3 activity

**Fig.5:**
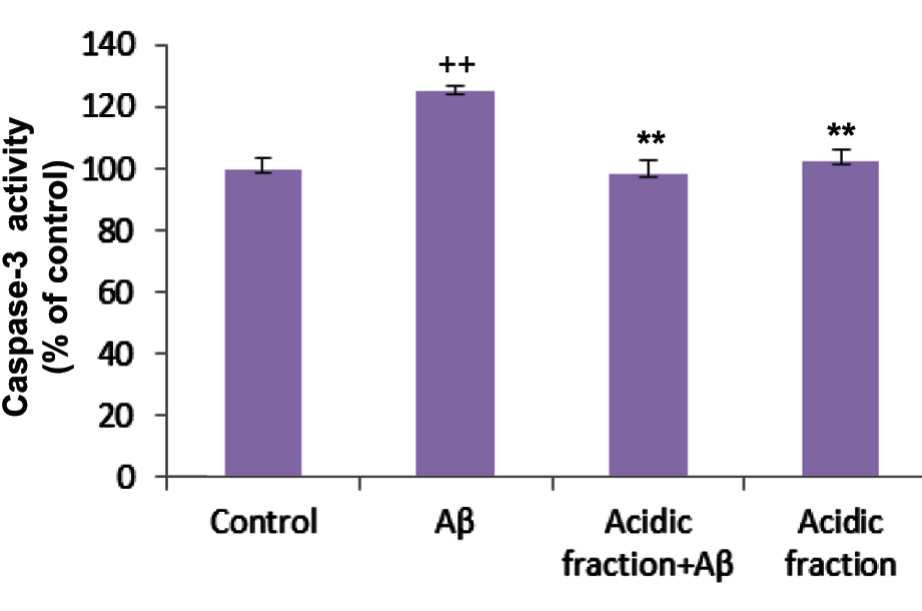
Caspase-3 activity in the studied groups. ++; P<0.01 versus control and **; P<0.01 vs. beta-amyloid (Aβ) treated.

The activity of caspase-3 in CGNs increased following exposure to 20 µM Aβ for 48 hours (Fig.5). However, pretreatment of CGNs with 30 µg/ml of the acidic fraction for 24 hours significantly reduced caspase-3 activity. 

### Hoechst/Annexin Vfluorescein isothiocyanate/ propidium iodide staining

**Fig.6:**
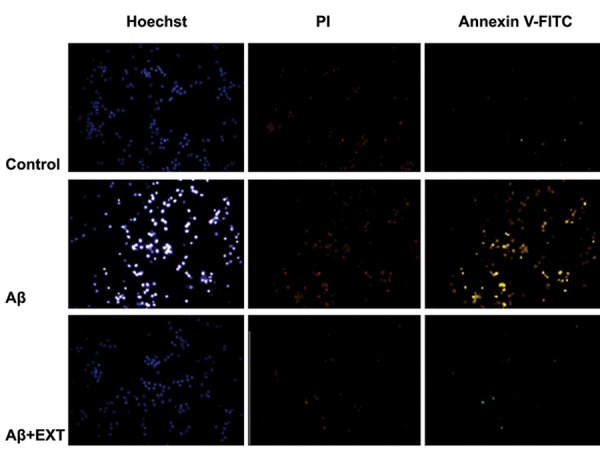
Results of Hoechst/annexin V-fluorescein isothiocyanate (FITC)/propidium iodide (PI) staining.

This staining was used for detection of apoptotic cells in the culture (Fig.6). Aβ-treated cells showed intense Hoechst staining because of DNA fragmentation in the apoptotic cells. Annexin V detected phosphatidylserine (PS) moieties which flip out from the inside of the cell membrane during apoptosis. The number of apoptotic cells stained with annexin V-FITC increased in Aβ-treated cells. PI staining indicated late apoptotic and necrotic cells that increased in Aβ-treated cells. Pretreatment with the acidic fraction of the extract decreased the numbers of apoptotic cells. 

## Discussion

In the present study our findings indicated that the acidic fraction of *M. officinalis* protected cultured CGNs from Aβ-induced toxicity. 

Mecamylamine, a nicotinic receptor antagonist, did not affect the protective effect of the acidic fraction. This finding showed that the protective effect of the acidic fraction of this extract could not be produced through the nicotinic receptors. 

Oxidative stress and apoptosis play crucial roles in Aβ-induced toxicity (4). The apoptotic processes were initiated by the addition of Aβ in neuron cultures (27). One possible mechanism for inducing apoptosis is production of ROS which results in lipid peroxidation and oxidative stress (28). Our results have indicated that pretreatment of CGNs with the acidic fraction attenuated Aβ-induced ROS production and lipid peroxidation. Caspase activity elevated by Aβ was reduced to the control level by the acidic fraction. Hoechst/annexin V/PI staining showed less numbers of cells with apoptotic morphology in the culture pretreated with the acidic fraction. These findings indicated that the acidic fraction of the extract attenuated oxidative stress and apoptosis. 

*M. officinalis* has potent antioxidant with direct free radical scavenging activity. This herb contains polyphenols which have potent antioxidant activities. Polyphenols are polar compounds that concentrate in the acidic fraction. It has been suggested that polyphenols in the acidic fraction are compounds that effectively protect CGNs against Aβ toxicity by anti-apoptotic and antioxidant activities. The polyphenols present in the *M. officinalis* extract are caffeic acid derivatives. Among them, rosmarinic acid is the major polyphenol component (29). 

Rosmarinic acid is a potent antioxidant with direct free radical scavenging activity. Neuroprotective activity is also reported for this compound. Rosmarinic acid has been shown to protect Aβ-induced memory impairment in mice which is due to direct peroxynitrite scavenging activity (30). Rosmarinic acid has anti-apoptotic activity. Rosmarinic acid could protect PC12 cells against Aβ-induced apoptosis by inhibiting caspase-3 activation, Aβ produced DNA fragmentation in PC12 cells, and by the p38 MAP kinase pathway which resulted in inhibition of tau protein hyperphosphorylation in PC12 cells (31). Evidence favors AD related mitochondrial dysfunction and apoptotic cell death (32). Rosmarinic acid has mitoprotective activity and inhibits permeation of mitochondrial membranes produced by Aβ aggregates. Rosmarinic acid ameliorates Aβ-induced apoptotic cell death by stabilizing mitochondrial membranes (33). Studies have shown that caspase-3 activation increased in AD patients. Activated caspase-3 colocalized with NFTs and senile plaques which suggested that caspase-3 has a role in synaptic failure during AD development (34,35). It is reported that rosmarinic acid is a potent caspase-3 inhibitor. Therefore it can halt apoptotic processes by inhibition of caspase-3 (36). In SHSY5Y cells, rosmarinic acid has been shown to attenuate H_2_O_2_-induced apoptosis by upregulation of the antioxidant enzyme heme oxygenase-1 (HO-1) through protein kinase A (PKA) and phosphatidylinositiol-3-kinase (PI3K) signaling pathways (37). Rosmarinic acid could prevent apoptosis in other tissue such as cardiac muscle (38), the kidneys (39), muscle cells (40), and the liver (41). 

The results of our study showed that Aβ increased AChE activity in CGNs. Pretreatment of CGNs with the acidic fraction of the extract could diminish the increased level of AChE activity. However we did not observe inhibition of AChE activity following pretreatment with the acidic fraction of the extract alone. This finding agreed with our previous study that indicated *M. officinalis* extract could protect PC12 cells against Aβ-induced cytotoxicity at a concentration lower than the concentration needed for AChE inhibition. Apart from catalytic activity, the AChE enzyme has noncatalytic activity and expression of this protein induced under stressful conditions such as oxidative stress (42). It was suggested that the acidic fraction of this extract could attenuate the increase in Aβ-induced AChE activity by alleviating oxidative stress. 

## Conclusion

The results obtained in this study indicated that the acidic fraction of *M. officinalis* extract ameliorated Aβ-induced oxidative stress and apoptosis. Nicotinic receptors were not involved in a protective activity of the extract. It was suggested that these effects of the extract could be attributed to the polyphenolic compounds, as well as their antioxidant and antiapoptotic activities. 

## References

[1] Prasansuklab A, Tencomnao T (2013). Amyloidosis in Alzheimer’s disease: the toxicity of amyloid beta (Aβ), mechanisms of its accumulation and implications of medicinal plants for therapy. Evid Based Complement Alternat Med.

[2] Hardy J, Selkoe DJ (2002). The amyloid hypothesis of Alzheimer’s disease: progress and problems on the road to therapeutics. Science.

[3] Friedlander RM (2003). Apoptosis and caspases in neurodegenerative diseases. N Engl J Med.

[4] Obulesu M, Lakshmi MJ (2014). Apoptosis in Alzheimer’s disease: an understanding of the physiology, pathology and therapeutic avenues. Neurochem Res.

[5] Eckert A, Marques CA, Keil U, Schussel K, Muller WE (2003). Increased apoptotic cell death in sporadic and genetic Alzheimer’s disease. Ann N Y Acad Sci.

[6] Sureda FX, Junyent F, Verdaguer E, Auladell C, Pelegri C, Vilaplana J (2011). Antiapoptotic drugs: a therapautic strategy for the prevention of neurodegenerative diseases. Curr Pharm Des.

[7] Anekonda TS, Reddy PH (2005). Can herbs provide a new generation of drugs for treating Alzheimer’s disease?. Brain Res Brain Res Rev.

[8] Zargari A (1992). Medicinal plants.

[9] Naghibi F, Mosaddegh M, Mohammadi Motamed M, Ghorbani A (2005). Labiatae family in folk medicine in Iran: from ethnobotany to pharmacology. Iran J Pharm Res.

[10] Alijaniha F, Naseri M, Afsharypuor S, Fallahi F, Noorbala A, Mosaddegh M (2015). Heart palpitation relief with Melissa officinalis leaf extract: double blind, randomized, placebo controlled trial of efficacy and safety. J Ethnopharmacol.

[11] Dobetsberger C, Buchbauer G (2011). Actions of essential oils on the central nervous system: an updated review. Flavour Fragr J.

[12] Dastmalchi K, Ollilainen V, Lackman P, Boije af Gennäs G, Dorman HJ, Järvinen PP (2009). Acetylcholinesterase inhibitory guided fractionation of Melissa officinalis L. Bioorg Med Chem.

[13] Pereira RP, Fachinetto R, de Souza Prestes A, Puntel RL, da Silva GNS, Heinzmann BM (2009). Antioxidant effects of different extracts from Melissa officinalis, Matricaria recutita and Cymbopogon citratus. Neurochem Res.

[14] Akhondzadeh S, Noroozian M, Mohammadi M, Ohadinia S, Jamshidi A, Khani M (2003). Melissa officinalis extract in the treatment of patients with mild to moderate Alzheimer’s disease: a double blind, randomised, placebo controlled trial. J Neurol Neurosurg Psychiatry.

[15] Soodi M, Naghdi N, Hajimehdipoor H, Choopani S, Sahraei E (2014). Memory-improving activity of Melissa officinalis extract in naive and scopolamine-treated rats. Res Pharm Sci.

[16] Sepand MR, Soodi M, Hajimehdipoor H, Soleimani M, Sahraei E (2013). Comparison of neuroprotective effects of Melissa officinalis total extract and its acidic and nonacidic fractions against A beta-induced toxicity. Iran J Pharm Res.

[17] Wake G, Court J, Pickering A, Lewis R, Wilkins R, Perry E (2000). CNS acetylcholine receptor activity in European medicinal plants traditionally used to improve failing memory. J Ethnopharmacol.

[18] Liu Q, Zhao B (2004). Nicotine attenuates beta-amyloid peptide-induced neurotoxicity, free radical and calcium accumulation in hippocampal neuronal cultures. Br J Pharmacol.

[19] Krämer D, Minichiello L (2010). Cell culture of primary cerebellar granule cells. Methods Mol Biol.

[20] Hajimehdipour H, Amanzadeh Y, Sadat Ebrahimi SE, Mozaffarian V (2003). Three tetraoxygenated xanthones from Swertia longifolia. Pharm Biol.

[21] Mosmann T (1983). Rapid colorimetric assay for cellular growth and survival: application to proliferation and cytotoxicity assays. J Immunol Methods.

[22] Williamsom KS, Hensley K, Floyd RA, Hensley K, Floyd RA (2003). Fluorometric and colorimetric assessment of thiobarbituric acid-reactive lipid aldehydes in biological matrices. Methods in pharmacology and toxicology: methods in biological oxidative stress.

[23] Bradford MM (1976). A rapid and sensitive method for the quantitation of microgram quantities of protein utilizing the principle of protein-dye binding. Anal Biochem.

[24] Ellman GL, Courtney KD, Andres V Jr, Feather-Stone RM (1961). A new and rapid colorimetric determination of acetylcholinesterase activity. Biochem Pharmacol.

[25] Wang H, Joseph JA (1999). Quantifying cellular oxidative stress by dichlorofluorescein assay using microplate reader. Free Radic Biol Med.

[26] Wu J, Jing L, Yuan H, Peng SQ (2011). T-2 toxin induces apoptosis in ovarian granulosa cells of rats through reactive oxygen species-mediated mitochondrial pathway. Toxicol Lett.

[27] Loo DT, Copani A, Pike CJ, Whittemore ER, Walencewicz AJ, Cotman CW (1993). Apoptosis is induced by betaamyloid in cultured central nervous system neurons. Proc Natl Acad Sci USA.

[28] Butterfield DA (2002). Amyloid beta-peptide (1-42)-induced oxidative stress and neurotoxicity: implications for neurodegeneration in Alzheimer’s disease brain.A review. Free Radic Res.

[29] Petersen M, Simmonds MS (2003). Rosmarinic acid. Phytochemistry.

[30] Alkam T, Nitta A, Mizoguchi H, Itoh A, Nabeshima T (2007). A natural scavenger of peroxynitrites, rosmarinic acid, protects against impairment of memory induced by Aβ 25-35. Behav Brain Res.

[31] Iuvone T, De Filippis D, Esposito G, D'Amico A, Izzo AA (2006). The spice sage and its active ingredient rosmarinic acid protect PC12 cells from amyloid-beta peptideinduced neurotoxicity. J Pharmacol Exp Ther.

[32] Friedland-Leuner K, Stockburger C, Denzer I, Eckert GP, Muller WE (2014). Mitochondrial dysfunction: cause and consequence of Alzheimer’s disease. Prog Mol Biol Transl Sci.

[33] Camilleri A, Zarb C, Caruana M, Ostermeier U, Ghio S, Hogen T (2013). Mitochondrial membrane permeabilisation by amyloid aggregates and protection by polyphenols. Biochim Biophys Acta.

[34] D'Amelio M, Cavallucci V, Middei S, Marchetti C, Pacioni S, Ferri A (2011). Caspase-3 triggers early synaptic dysfunction in a mouse model of Alzheimer’s disease. Nat Neurosci.

[35] Louneva N, Cohen JW, Han LY, Talbot K, Wilson RS, Bennett DA (2008). Caspase-3 is enriched in postsynaptic densities and increased in Alzheimer’s disease. Am J Pathol.

[36] Khan S, Ahmad K, Alshammari EM, Adnan M, Baig MH, Lohani M (2015). Implication of caspase-3 as a common therapeutic target for multineurodegenerative disorders and its inhibition using nonpeptidyl natural compounds. Biomed Res Int.

[37] Lee HJ, Cho HS, Park E, Kim S, Lee SY, Kim CS, et al (2008). Rosmarinic acid protects human dopaminergic neuronal cells against hydrogen peroxide-induced apoptosis. Toxicology.

[38] Li XL, Liu JX, Li P, Zheng YQ (2014). Protective effect of rosmarinic acid on hypoxia/reoxygenation injury in cardiomyocytes. Zhongguo Zhong Yao Za Zhi.

[39] Domitrovic R, Potocnjak I, Crncevic-Orlic Z, Skoda M (2014). Nephroprotective activities of rosmarinic acid against cisplatin-induced kidney injury in mice. Food Chem Toxicol.

[40] Chen KL, Li HX, Xu XL, Zhou GH (2014). The protective effect of rosmarinic acid on hyperthermia-induced C2C12 muscle cells damage. Mol Biol Rep.

[41] Domitrovic R, Skoda M, Vasiljev Marchesi V, Cvijanovic O, Pernjak Pugel E, Stefan MB (2013). Rosmarinic acid ameliorates acute liver damage and fibrogenesis in carbon tetrachloride-intoxicated mice. Food Chem Toxicol.

[42] Hartl R, Gleinich A, Zimmermann M (2011). Dramatic increase in readthrough acetylcholinesterase in a cellular model of oxidative stress. J Neurochem.

